# How to Internationalize Medical Education using Concepts in Internationalization of Higher Education

**DOI:** 10.15694/mep.2020.000151.1

**Published:** 2020-07-27

**Authors:** Anette Wu, Geoffroy Noel

**Affiliations:** 1 Department of Pathology and Cell Biology; 2 Department of Anatomy and Cell Biology and Institute of Health Sciences Education

**Keywords:** Internationalization of medical education, interdisciplinary approach, program analysis, medical school curriculum, innovative methods

## Abstract

This article was migrated. The article was marked as recommended.

Internationalization of *higher* education is a well-researched area with a long history. At a time of increasing globalization, particularly in light of recent global health events, the internationalization of *medical* education may play an important role in medical school teaching - increasing future medical collaboration and building a global medical community. Internationalization of medical education is a less researched discipline and often limited to areas of Global Health. It is typically not part of the standard medical curriculum. While internationalization of medical education has overlapping themes with Global Health education, it has a much wider scope (i.e., preparation of global citizen physicians, international employability, collaboration, cultural and international understanding).

Lessons learned from concepts in internationalization of higher education can aid in the realization of internationalization of medical education programs and establish IoME as a distinct area of educational research.

This paper suggests elements to consider when implementing programs in internationalization of medical education. Guided by the analysis of an existing program in internationalization of medical education, important components are highlighted from the perspective of concepts found in internationalization of higher education.

Several elements that are important features in internationalization of higher education are emphasized - institutional partnerships, goal setting, variety of internationalization at home concepts, international classroom features, multi-directional student mobility, and sustainability.

The authors aim to shed light on the area of internationalization of medical education, widen its scope from Global Health education, and introduce it as a field of study for educational research. Adapting models and concepts of international higher education can help with establishing this field.

## Introduction

In the November 2019 issue of the MedEdWorld Newsletter of the AMEE (“An International Association of Medical Educators”) Dr. Ronald Harden reflected in his blog on the internationalization of *higher* education (IoHE) and developing students’ global-international and inter-cultural competencies ( Harden, 2019). Inspired by this publication, we investigated how to connect the area of IoHE with the internationalization of *medical* education (IoME) and how to help others work in IoME as an area of educational research.

IoHE is defined as “..the conscious effort to integrate and infuse international, intercultural, and global dimensions into the ethos and outcomes of postsecondary education..” ( Hudzik, 2015).

Historically, research on IoHE has been a domain of the social sciences ( Knight, 2012; De Wit *et al.*, 2015; Hudzik, 2015; Altbach, 2016). IoHE is complex and motivations for IoHE include academic, socio-cultural, political, and economic goals ( De Wit, 1998; Hudzik, 2015) - resulting in a major impact in these areas.

IoME, the *medical* counterpart of IoHE, does not have a formal definition. While some use “the process of purposefully integrating international, intercultural, or global dimensions into medical education in order to enhance its quality and prepare graduates for professional practice in a globalised world” to describe it - IoME is not a defined field. As an educational research field it is less studied and often work is found within publications of other health professions - i.e. Nursing and Public Health research ( Yarbrough, 2014; Green and Whitsed, 2015) - although sporadic reports exist ( Knipper *et al.*, 2015; Stütz *et al.*, 2015).

At a time of global interconnectedness, particularly in light of recent global health events (i.e., the SARS and COVID-19 pandemics), IoME can prepare future physicians to practice with a global frame of reference and to help with future international collaborations. IoME can lead to enrichment of medical education by enhancing medical students’ understanding of social, cultural, and ethical differences - thus, preparing them to be part of a global medical community, and leading to global-minded and socially accountable physicians. IoME can therefore have a major impact on the improvement of healthcare - both locally and globally.

IoME should not be equated with *Global Health* education ( Yarbrough, 2014). Global Health is a term frequently used to refer to the research and practice of health in the low and middle income countries - although the generally accepted definition has a much wider scope ( Koplan *et al.*, 2009). In the global body of literature, particularly in the US, the term Global Health as currently used, is often used as a proxy for IoME. However, while there is overlap between Global Health and IoME, these two areas are distinct. Originally rooted in the domain of Public Health, Global Health education has mostly focused its educational goals on health equity and social justice - addressing issues revolving around low and middle income countries and/or underserved populations ( Yarbrough, 2014). IoME, while related to Global Health, is a much broader term and includes consideration of how intercultural and international factors might impact professional practice and medical education locally. IoME impacts international understanding, preparation for international employability, and leads to a global medical world. Educational goals between the two areas (i.e., teaching of cultural sensitivity, learning about different healthcare systems and approaches, globalization of education, international experiences, career enhancement) can overlap.

As with IoHE, IoME should never be regarded as a goal itself, but it is to serve as a means to a specific educational goal in healthcare ( De Wit, 2011).

One of the goals in IoHE is the preparation of global citizen graduates who will have an impact on social and economic advancement in the world ( Hudzik, 2011). It is expected that with increased globalization international health workforce exchanges and global exchange of patients/patient care will increase - thus making IoME an important element of students’ education. Research in IoME is needed in order to find best practices to prepare the next generation of physicians for these global challenges and could aid in making curricular changes within medical schools.

Similar to IoHE, IoME may have different meanings and goals for different medical schools and countries ( Hudzik, 2011). In the “market model”, medical schools aim to be competitive in the global market - for themselves and for students. In the “liberty” model, which was introduced in the post WWII era, medical schools aspire to support and maintain world peace via international understanding and collaboration. In the “social justice” model, medical schools emphasize humanitarian work to help with issues regarding health equality and Global Health - at home and globally ( Warner, 1992; Hanson, 2015).

Global competencies in IoHE often aim to educate global citizen graduates with the goal of international employability. Global competencies in IoME are less well defined and more complex, including areas of Global Health and overlapping with areas of Public Health. To date, global competencies in IoME are not agreed upon, and internationalization programs vary without official guidelines or agreed upon formats ( Jeffrey *et al.*, 2011; Khan *et al.*, 2013; Yarbrough, 2014).

Similar to IoHE, IoME can be achieved on many levels in academia ( Harden, 2006; Hudzik, 2015). It can include institutional levels within a university, faculty, and students ( Qiang, 2003; Knight, 2012). On the student level it can include curriculum and extracurricular activities, student inbound and outbound mobility.

Concepts and lessons learned from studies in IoHE could be helpful in endeavors involving the realization of IoME.

In this publication we suggest what elements should be considered for implementing work in IoME. These elements are drawn from the last 25 years of published concepts in IoHE. We base our suggestions on an analysis of the componentss of an existing IoME program that is founded on a collaboration of 14 international universities - two North American, eight European, three East Asian, and one Australian medical school ( Wu *et al*. 2020). Goals, concepts, and frameworks used in IoHE are applied to the elements of this international medical education program as examples.

This publication is not meant to re-present details of an existing program. It is a focused analysis of a program in IoME developed via a much needed connection to IoHE - thus, bringing two fields of educational research closer together so that other medical educational researchers can learn from our experiences and use them as a guide for future endeavors. We hope to contribute to the body of literature on educational research about IoME, to emphasize similarities and differences between IoHE and IoME, and to encourage other medical educators to analyze their international efforts in light of goals, experiences, and concepts drawn from studies in IoHE.

In order to fully appreciate the analysis a brief summary of the program is initially presented.

## Methods

### The “International Collaboration and Exchange Program”

In 2014, faculty members of the Pathology department in the medical school at Columbia University, New York, USA, initiated an IoME program with the mission of enhancing the education of the next generation of global healthcare leaders. The program goal was to prepare preclinical medical and dental students for future global healthcare leadership roles. The above goal centered on the idea that future healthcare leaders are expected to solve healthcare problems in an international collaborative manner. Therefore, it was felt that student education had to include a well-structured global component inside and outside the classroom.

Via offerings of a faculty-led platform for student networking and exchange, the program included international small-group online work covering international healthcare related topics over the course of one semester. The above was followed by group presentations at international student conferences, and subsequent multi-directional basic science rotations in an international research laboratory. Initially intended as a small pilot project between a few selected universities, it developed into a full internationalization program ( Wu *et al*., 2019). Program elements aimed to provide preclinical medical and dental students with the skills for international leadership via early exposure to their international peers, intercultural exchange, and by familiarizing them with medical systems and health related topics in other countries. Learning about Public Health, cultural exchange between students’ via online contacts, immersion into a different academic life abroad, and internationalizing the home campuses were also goals.

In this program IoME was not considered the end goal. IoME rather served as a means to foster global-minded physicians and oral health physicians.

To provide a framework for long-term sustainability the start of the program was deliberately anchored at the beginning of the preclinical portion of medical school.

Uniquely, this program was founded by the collaboration of multiple international anatomy departments. The rationale behind connecting an educational discipline that is seemingly far removed from international education is that anatomy education is found in every medical curriculum and is taught early in all medical schools. The discipline of anatomy is tightly connected with student education and the basic sciences, making it an easy access point to reach students, and can be used as a commonality for students to connect with each other.

Over the course of five years, we successfully established a network of 14 international universities on four continents, which provided the foundation for the students’ early international exposure ( Table 1).

**Table 1.** Partner Universities

Partner University	City, Country
The University of Sydney	Sydney, Australia
Medical University of Vienna	Vienna, Austria
McGill University	Montreal, Canada
University of Copenhagen	Copenhagen, Denmark
University of Helsinki	Helsinki, Finland
University of Paris Descartes	Paris, France
Ludwig Maximilians University	Munich, Germany
Martin Luther University	Halle-Wittenberg, Germany
Kyoto University	Kyoto, Japan
Tokyo Women’s Medical University	Tokyo, Japan
National Taiwan University	Taipei, Taiwan
King’s College London	London, United Kingdom
University of Cambridge	Cambridge, United Kingdom
Columbia University	New York, United States of America

We recently shared more program details and qualitative outcomes of student learning within this program ( Wu *et al*., 2020)

### Analysis of the Program

Our analysis was based on concepts in IoHE, recommendations from the Association of International Educators (NAFSA) ( Hudzik, 2011) and Association of International Education Administrators, and publications by researchers in IoHE ( Qiang, 2003; De Wit, 2011; Ozturgut *et al.*, 2014; Hudzik, 2015) (see Table 2).

## Results

The elements detailed below were identified from research in IoHE and adapted into our program ( Table 2). They were deemed to be important for the success and sustainability of the program.

**Table 2.** Elements of IoHE identified in IoME Program

Element of IoHE	Represented in Program
International partnerships/collaborations	Collaboration of anatomy departments at international universities
Accessibility	Voluntary program for all medical and dental students
Internationalization at home	International classroom topics, international online small-group work, international online conferences, international students
Student mobility	Multi-directional short-term student exchanges
Leadership	Multi-layered leadership (local and international students and faculty, university leadership)
Academic unit	Anatomy departments
Specific goals	Supporting the next generation of global healthcare leaders
Sustainability	Low cost, dedicated faculty, student involvement, evaluation, innovation and adaptability, legacy and community, beyond the program activities
Integration into institutional processes and vision	Shared vision and goals of anatomy faculty members and institutions

### International institutional partnerships

In IoHE international academic partnerships and collaborations are encouraged and important. Limiting the number of institutional partners is highly recommended ( De Wit, 2011; Deardorff and Charles, 2018). While a specific number is not suggested, a “more is better” attitude is discouraged. In our opinion this approach is applicable and important for IoME - particularly for programs that are overseen by individual faculty members with limited time and administrative resources. Partners should be carefully selected based on shared visions and goals. Most importantly, the partnership should serve all partners. We limited the number of partners to 14 in order to fully dedicate time and resources to each partner. Because of the limited number, a careful preselection process took place. International partnerships were initiated via anatomy faculty utilizing a systematic selection process considering quality of research opportunities, culturally rich location, high percentage of English-speaking students, safety for student travel, and other criteria. We selected partners on four continents, from similar economic and academic backgrounds but sufficiently culturally diverse for junior students to appreciate the differences. Finally, it is important to limit number of partners at the beginning of new programs.

### Accessibility

One key component of IoHE is general accessibility ( Hudzik, 2011). We recommend general accessibility of internationalization programs to all eligible students. Because of the nature of our program the participation was limited to medical and dental students. However, the program was voluntary, tuition free and without fees, and therefore accessible to all preclinical students of the respective partner schools. Our target group was students with academic and leadership aspirations. In order to manage student numbers locally some schools opted for a pre-selection process (i.e., exam grades, interest in the basic sciences, interest in an academic career path, interest in international healthcare, propensity towards leadership). Of note, the inclusion of dental students as future oral healthcare leaders broadened accessibility for healthcare professionals. More recently, the program has been open to preclinical college students from the network schools who aspire to attend medical school.

### Internationalization at home

The components of internationalization at home play an increasingly important role in IoHE ( Leask, 2009; Leask, 2013; Beelen and Jones, 2015). Reasons for this include resource efficiency and general accessibility. It can include format, global teaching content, and students. In medical education, internationalization at home has only recently become a focus ( Liauw *et al.*, 2018). To reach a large number of healthcare students at home this approach should be developed and expanded in the future and educational research in this area is needed.

#### Internationalization in the classroom

Classroom internationalization in higher education can include the content of teaching material (i.e., language, global educational content, cultural competency), online work, and an international student body ( Qiang, 2003).

We recommend a variety of internationalization at home elements in order to achieve different learning objectives. In our program we included the following international classroom elements.

##### International small-group peer-to-peer online work

Contact with international peers is one important element in IoHE. While typically this is achieved via student mobility, in our program the online portion gave students a platform to initially connect with peers without traveling. Students practiced international collaboration and cultural exchange online, across borders. Current world events demonstrate how important, efficient, and safe, virtual, non-physical interaction can be. Thus, this element should be considered when planning IoME programs.

##### Intercultural exchange at home

Acquisition of cultural competency is an important goal of IoHE ( Hudzik, 2011). While most international program goals include cultural competency as a soft skill, cultural awareness was deliberately included as a distinct part of our program. We recommend separate formal awareness building of cultural differences, because students at this early stage in life often have limited knowledge about the culture of other countries.

In our program students initially compared the structure of their anatomy courses, and they reflected on topics related to body donation (e.g., appreciation of life and the donor, life’s passing, etc.). Because there is cultural variation in regard to these topics, this exercise offered the opportunity for a cultural learning experience.

Furthermore, at two student conferences at the end of the semester each country presented their culture to the student cohort (e.g., history, art, architecture, literature, geography, food, etc.), in order to deepen cultural understanding for each other.

##### Global content at home

Global educational content is a desired element in IoHE ( Hudzik, 2011) and applies to IoME as well.

Unlike IoHE, global content in medical education can include specific topics in the pathology and treatment of diseases (e.g., tropical diseases), or general public health related topics. In our program we focused on areas that are not typically covered in a medical curriculum, in order to give students insight into similarities and differences in international public health.

The student groups discussed and compared their health education and delivery systems, and health ethics and laws. Subsequently, they wrote a summary paper regarding global public health challenges and compared different approaches to addressing these problems in each country.

Awareness of international differences in these areas is crucial knowledge for global citizen physicians who engage in scientific research and international collaboration and enables students to feel that they are part of a larger medical community.

##### International online student conferences

Participation in international activities. In our opinion international activities can provide a good exercise to practice tolerance of international peers and foreign customs. After completion of the small group work all students participated in two large online conferences to present their work to each other, and to practice international data presentation. The conferences were moderated by students and were overseen by faculty. We recommend this exercise in early international presentation, which provides additional desired international skills.

#### Internationalization of the campus

##### Inbound student mobility

For a long time the number of enrolled international students was considered a determinant for success of IoHE ( Hudzik, 2011). However, this has been recently questioned ( De Wit, 2011). While we agree that student mobility as a sole international activity restricts the number of participating students to those who can afford to engage in international projects, up to now student mobility has been a viable option to engage students in international activities. This element of IoME will remain an important component of international programs.

In this program integrating short-term international students on campus offered an efficient and innovative way to internationalize the campus - avoiding high tuition and elaborate administrative visa paperwork.

Program participants, including those who opted not to travel, hosted the incoming students, and invited classmates that were not part of the program to mingle. There were several social activities per week and every weekend had planned activities - organized by the host students and faculty. Although not all students can afford to travel, in our experience a second opportunity for peer interaction increases the likelihood of long-term sustainability. We are currently investigating whether the second peer interaction can also be achieved via an online program (work in progress).

##### Outbound student mobility

IoHE work recommends that student international internships/research abroad should be embedded as part of a larger picture and not just a mere abroad program ( Hudzik, 2011). For medical students, research abroad can be considered a career enhancer, but for IoME it serves the additional aforementioned educational goals. We recommend multi-directional travel in order to appreciate the full spectrum of cultural exchange and for students to experience being both hosts and visitors.

In the semester following their online work students joined a basic sciences laboratory in one of the partner countries, enhanced their intercultural experience, and immersed themselves in a different academic life abroad. The travel was multi-directional and staggered into four travel groups, to allow for overlap of local and incoming students.

Students subsequently remained in contact via social media - thus internationalization continued beyond the campus after the program ended. Social media, overseen by the program, is an important means to maintain the long-term effects of the program.

### Elements for success

Success of IoHE programs is highly dependent on shared values and visions, on leadership, and stakeholder levels ( Hudzik, 2015; Deardorff and Charles, 2018). Below are key elements found in IoHE, and featured prominently in our program, that were thought to be crucial for the success of this IoME program.

#### Integration into a discipline

In contrast to IoHE, in medical education there is no defined discipline that focuses on internationalization. One key element of the program was its integration into an educational discipline within medicine. We uniquely based our international efforts upon the leadership of *anatomists*, in a medical discipline that is known to teach “soft skills” (e.g., teamwork, collaboration, professionalism, empathy, etc.) that go beyond the pure teaching of anatomy facts. Advantages of involving anatomy in internationalization efforts are: 1) anatomy is taught at every medical school, 2) anatomy faculty have access to young, preclinical students (most are in their formative years of life), 3) anatomists are involved with education; typically, with dedicated teaching time, 4) anatomists are linked to the basic sciences, and 5) each school has only a few, easily identifiable anatomists.

We highly recommend innovative integration into a discipline. Historically, some disciplines (e.g., Infectious Diseases) were the primary focus of international work. We prefer a discipline that is easily accessible to all students and not necessarily historically typical of international work. Interdisciplinary approaches with other departments and schools (e.g., East Asian Studies) are also viable options.

#### Involvement of university leadership

We recommend close communication with university leadership in order to improve efficiency and transparency. Often, international medical programs are based on activities led and conducted by individual faculty members. Support by academic administration and senior leadership, even for programs initiated by an individual faculty member, remains an important element of IoME.

Despite the extracurricular nature of our program, regular communication with general university service units such as the deans for education, student affairs, research, and international programs, ensured that university officials were kept informed of the progress and success of the program. In addition, those units played a crucial role in executing the official legal paperwork for school partnerships.

#### Student leadership

Student leadership is important and should always be included in IoME programs, as it is recommended in IoHE (see also “sustainability”).

Each school provided at least two local student leaders to ensure proper communication and visibility of the program. The formation of student leader groups provided a second layer of leadership - locally, and on the student level. This format had two purposes - to support students’ input into the format of the program, and to provide additional student leadership training.

#### Securing Sustainability

Sustainability in IoHE programs is important because programs and curricula need time to be built and successes are not immediately visible. The current program remained sustainable for a variety of reasons.

*Low cost.* Resource allocation for IoHE programs is complex and challenging ( Hudzik, 2015). Sustainability appears to be crucial for many IoME programs because of their extracurricular nature. The present program did not incur program costs. Faculty saw the potential for scholarly work and dedicated their uncompensated time to support the operations of the program (e.g., recruitment, organizing the conferences, student internship placements, etc.). Student travel was funded from different sources. Many students were self-funded.

*Dedicated faculty and involvement of students - the bottom up approach.* In IoHE often programs are supported by the institution from the top down. This approach can be challenging if faculty members’ visions and goals are not in line with the university’s goals. For many programs in IoME the reverse is true - programs are often initiated and driven by faculty and students. In this program faculty and students had a vested interest in the success of the program and were closely involved from year to year. Giving participants a role and independence are important factors in ensuring long-term sustainability.

*Program evaluation and research.* Regular evaluation of the program is important to keep the program up to date with students’ desires and goals. Every year data from the program was used for educational research. Scholarly work is not only an important means to keep faculty engaged but is necessary to improve the field of IoME.

*Adaptability and innovation.* IoHE has a long history and is an ever-evolving field ( Hudzik, 2015). Adaptability and the willingness to change are important elements for IoME. An annual questionnaire was sent to participants of this program. Changes were made based on their suggestions. Faculty members met at least twice during the program year to discuss potential upcoming pitfalls, problems, and innovations.

*Legacy and community.* Students took great pride in helping with recruitment of the next generation of student participants, helped with accommodation arrangements for incoming students, organized social activities, worked on the program website, and connected via social media. Information was passed on from year to year.

*Beyond the program.* In order to reach the goal of an international medical program, student engagement beyond the formal portion of the program is important. We recommend that program initiators have a long-term plan in mind so that the program extends beyond its formal portion. Our program’s goal was to prepare future global medical leaders. Students remained active beyond the length of formal student participation because of the program’s active website and presence on soical media. The students joined a life-long professional network group in LinkedIn ^©^ to remain connected as they moved on with their careers.

## Discussion

In the current article we provide suggestions, and describe the analysis of an IoME program from the perspective of research done in IoHE. We only focused on selected aspects of IoHE that were helpful and identifiable in the presented program - so medical educators can learn from experiences drawn from research in IoHE. Individual schools and programs may have to adapt their needs and draw from other components found in the vast literature of experiences found in IoHE.

Our publication is intended as motivation for medical educators to work on IoME. The authors also hope to inspire others to reflect on the topic of IoME as a field of study.

IoHE is a response of educational institutions and disciplines to the globalization of the world ( Hudzik, 2015; Deardorff and Charles, 2018). Research in IoHE indicates that internationalization has a major impact not only on student and university campus life, but is increasingly reflected in the institutions’ values, visions, and partnerships ( Hudzik, 2011; Hudzik, 2015). Therefore, alignment of values is very important. In order for IoME to move forward as a discipline and field of study, shared goals and objectives, consistent standard formats, and common values are therefore crucial. Our program was based on the shared goals of international anatomy departments, but overarching, shared institutional standards will need to be developed.

To date, most major medical academic institutions in the US and other industrialized countries aim towards, or are tightly involved with international research partnerships. It appears that despite these internationalization efforts in science and technology, IoME outside of Global Health has not been a major focus at many institutions. Our program did not concentrate on aspects of Global Health education, although a global impact on healthcare can be expected. We do not discount the importance of Global Health education in medical education but hope to broaden aspects of Global Health education to include comprehensive IoME.

In order to bring awareness of global aspects to medicine, IoME needs to find its place in medical school curricula. One might raise the concern that medical curricula currently are tightly packed, leaving little space for additional curricular disciplines to be introduced. We argue that with expected upcoming changes in healthcare (e.g., increased globalization, the advent of artificial intelligence, etc.) medical curricula will likely undergo major adjustments. This may represent a window of opportunity to revisit IoME as a curricular and not extracurricular activity in medical education - with agreed upon standards and goals. In the current health climate, this need seems more important than ever.

For many institutions student mobility currently serves as a synonym for IoME. In IoHE it was recently questioned whether mobility is a sufficient approach ( De Wit, 2011). In medical education (especially in the Anglo-Saxon schools) outbound mobility activities are often unsupported extracurricular activities - driven by students’ desires to go abroad. We feel that during a time of raised awareness of sustainability and carbon footprint, and more recently of safety concerns, one needs to take a step back and revisit whether student mobility without agreed upon and defined educational outcomes goals is the most efficient and justifiable approach. Unlike other student exchange programs ( IFMSA, 2019) our program is a multi-directional exchange program involving pre-departure peer-to-peer work with students from different parts of the world ( Ten Cate and Durning, 2007) - thus, ensuring intercultural exchange prior to travel. Pre-departure awareness of cultural differences is considered an ethical requirement for student travel and promotes sustainability.

In addition to student mobility, innovative methods and research regarding how to introduce internationalization at home as it is currently done in IoHE is strongly recommended ( Beelen and Jones, 2015).

We highlight a small portion of what is possible within IoME as an educational discipline and as a field of study. Research on IoHE has been in place for decades. IoME will have to establish its role and find resources to develop.

The complexity of IoME should not be underestimated. According to Hudzik and Deardorff & Charles all aspects of higher education can be affected by internationalization ( Hudzik, 2011; Hudzik, 2015; Deardorff and Charles, 2018) - and so it will be for IoME. Resources and structures need to be set in place in order to provide comprehensive IoME.

Also, in contrast to IoHE, there is no field in medicine that prepares medical *educators* to teach international topics. Few have gotten additional training (i.e. in Global Health, via Schools of Public Health), or are involved with international humanitarian work. Research is needed to evaluate how faculty training in international teaching and international scholarly work can be introduced. Collaboration with the social sciences and using concepts of IoHE will be of help in establishing this field.

## Limitations

We only touched upon the vast amount of published research on IoHE. Cited references are not meant to be comprehensive. Our purpose is not to provide a summary of literature on IoHE, but to give recommendations based on our experiences, via reflecting on one program and comparing it to what has been done in other disciplines - for others to learn, in order to efficiently plan and execute IoME programs as the field develops.

A limitation of our recommendations based on our program analysis is its perspective from the view of high-income countries (in particular, the US). To be inclusive of all medical schools, views from programs and countries of different backgrounds need to be included to further establish the field of IoME.

## Conclusion

International programs should be established and analyzed in view of existing concepts of IoHE. Lessons learned in IoHE can help to efficiently plan and execute comprehensive IoME programs. Research in IoME as a field of study is needed in order to establish guidelines to best prepare global healthcare professionals and to prepare medical teachers.

Our recommendations and analysis are not meant to recap the development or the description of a program but are meant to serve as an analysis of its format in order to draw a connection to IoHE; to aid other educators establishing programs in IoME build on experiences from IoHE.

## Take Home Messages

• IoME is important at this time of globalization and should be included in medical school curricula.
• Concepts learned from experiences in IoHE can help with efficiently planning and executing comprehensive IoME programs.
• Collaborative and interdisciplinary approaches are important.
• Research in IoME as a field of study is needed in order to establish goals, guidelines, and standards, to best prepare healthcare professionals for working in a global medical world and to optimally prepare medical teachers.

## Notes On Contributors

Anette Wu, MD, MPH, PhD, is an assistant professor in the Department of Pathology and Cell Biology, Vagelos College of Physicians and Surgeons, Columbia University, New York, NY, USA. She is the director of the International Collaboration and Exchange Program at Columbia University. ORCID: https://orcid.org/0000-0001-7341-7200

Geoffroy P.J.C. Noel, PhD, is an associate professor and the director of the Division of Anatomical Sciences, Department of Anatomy, Faculty of Medicine and Health Sciences Education, McGill University, Montreal, Canada. ORCID: https://orcid.org/0000-0003-2192-7756
