# Supplementary material for: How to Internationalize Medical Education using Concepts in Internationalization of Higher Education
Source: MedEdPublish (2016). 2020 Jul 27;9:151. [Version 1] doi: 10.15694/mep.2020.000151.1 (PMC10702653; doi:10.15694/mep.2020.000151.1)
Supplement: Peer review history

# Reviewer report: Reviewer response for version 1

**DOI:** 10.21956/mep.20042.r30690

**Published:** 2020-08-04

**Authors:** Jennifer Cleland

**Affiliations:** 1 University of Aberdeen

This review has been migrated. The reviewer awarded 4 stars out of 5 This is an interesting article, and I like the way the authors have draw on education more generally to inform innovative practice in medical education (anatomy education more specifically). The article is quite descriptive, and I would have liked to have seen more about what didn't work or wasn't useful, as that would have been just as informative as the positive outcomes. I would have also liked to see more reflection on the potential downsides of internationalisation, the issues of dominant west/north, colonialism and so on. This is touched on as a limitation (the recommendations are from the perspective of a high-income country, but is worthy of more consideration in relation to this topic.

Reviewer Expertise:

NA

No decision status is available

# Reviewer report: Reviewer response for version 1

**DOI:** 10.21956/mep.20042.r30692

**Published:** 2020-07-31

**Authors:** Megan Anakin

**Affiliations:** 1 University of Otago

This review has been migrated. The reviewer awarded 4 stars out of 5 I read this article with interest because I am interested in better understanding this topic. To help the reader better appreciate the ideas presented, the authors may wish to specify who the “some” are that offer the definition of internationalisation of medical education in the fourth paragraph of their introduction and presented a few brief examples of how it has been studied to help support the statements made in the fifth and sixth paragraphs. The authors build a warrant for the need to better understand the elements of internationalisation of medical education and their analysis of the literature. A programme is described to illustrate the elements. To better understand how the programme was analysed, please consider explaining the steps used to identify the components of the programme and how they were compared to or developed from the recommendations from NAFSA. Thank you for writing this thought-provoking practical tip/guideline article.

Reviewer Expertise:

NA

No decision status is available

# Reviewer report: Reviewer response for version 1

**DOI:** 10.21956/mep.20042.r30691

**Published:** 2020-07-27

**Authors:** mariam asaad amin ibrahim

**Affiliations:** 1 ain shams faculty of medicine

This review has been migrated. The reviewer awarded 5 stars out of 5 An amazing article, i am proud as an anatomist to see that anatomy curriculum is far used beyond the blunt teaching experience that we are used to.. i have always seen basic sciences as the first contact between faculty and students and that it would be very beneficial if we as basic scientists make advantage of this.. i will sure share this article idea with my professors to see if we can apply this in our institution ..Thank you

Reviewer Expertise:

NA

No decision status is available
